# Selinexor, a selective inhibitor of nuclear export, inhibits human neutrophil extracellular trap formation *in vitro*

**DOI:** 10.3389/fphar.2022.1030991

**Published:** 2022-11-24

**Authors:** Szilvia Baron, Tami Rashal, Dmitry Vaisman, Ronit Elhasid, Rachel Shukrun

**Affiliations:** ^1^ Pediatric Hemato-Oncology Research Laboratory, Tel Aviv Medical Center, Tel Aviv, Israel; ^2^ Sackler School of Medicine, Tel Aviv University, Tel Aviv, Israel; ^3^ Karyopharm Therapeutics Inc., Newton, MA, United States; ^4^ Department of Pediatric Hemato-Oncology, Tel Aviv Medical Center, Tel Aviv, Israel

**Keywords:** neutrophil, NETs formation, inhibition, selinexor, inflammation

## Abstract

Neutrophils are central players in the innate immune system. To protect against invading pathogens, neutrophils can externalize chromatin to create neutrophil extracellular traps (NETs). While NETs are critical to host defense, they also have deleterious effects, and dysregulation of NETs formation has been implicated in autoimmune diseases, atherosclerosis and thrombotic conditions, cancer progression and dissemination, and acute respiratory distress syndrome. Here, we report that selinexor, a first-in-class selective inhibitor of nuclear export approved for the treatment of multiple myeloma and diffuse large B-cell lymphoma, markedly suppressed the release of NETs *in vitro*. Furthermore, we demonstrate a significant inhibitory effect of selinexor on NETs formation, but not on oxidative burst or enzymatic activities central to NETs release such as neutrophil elastase, myeloperoxidase or peptidyl arginine deiminase type IV. The inhibitory effect of selinexor was demonstrated in neutrophils activated by a variety of NETs-inducers, including PMA, TGF-β, TNF-α and IL-8. Maximal inhibition of NETs formation was observed using TGF-β, for which selinexor inhibited NETs release by 61.6%. These findings pave the way to the potential use of selinexor in an effort to reduce disease burden by inhibition of NETs.

## Introduction

Neutrophils, the most abundant circulating leukocyte in the blood, are the first line of immune defense within the innate immune system (Cheng and Palaniyar, 2013). Neutrophils protect the host by several mechanisms including phagocytosis, release of cytotoxic molecules, and formation of neutrophil extracellular traps (NETs) (Malech et al., 2014). NETs are formed by activated neutrophils and are composed of DNA fibers, histones, and antimicrobial proteins (Brinkmann et al., 2004; Sollberger et al., 2018). However, it was recently demonstrated that NETs also form in non-infectious conditions. In fact, dysregulation of NETs formation or clearance has been associated with a wide variety of diseases including autoimmune diseases such as vasculitis, rheumatoid arthritis, systemic lupus erythematosus (Kessenbrock et al., 2009; Lee et al., 2017); atherosclerosis and thrombosis-associated conditions (Megens et al., 2012); cancer progression and dissemination (Masucci et al., 2020); and acute respiratory syndrome (ARDS) (Mikacenic et al., 2018), including lung injury caused by COVID-19 (Middleton et al., 2020). The involvement of NETs in the above pathologies spurred the search for inhibitors of NETs formation. Proposed agents target molecules involved in signal transduction and cell machinery involved in NETs release, such as elastase (NE), myeloperoxidase (MPO), peptidyl arginine deiminase type IV (PAD4) and gasdermin D (GSDMD).

Selinexor is a first-in-class selective inhibitor of nuclear export (PubChem, 2004). It is an orally available small molecule inhibitor of exportin-1, XPO1, chromosome region maintenance 1 protein (CRM1) which modifies XPO1-cargo-binding residue cysteine-528, thereby irreversibly inactivating XPO1-mediated nuclear export. Inhibition of shuttling of proteins such as tumor suppressors and growth regulatory proteins, can restore endogenous cell cycle arrest and selectively eliminate neoplastic cells (PubChem, 2004). Selinexor was recently approved for the treatment of multiple myeloma (MM) and diffuse large B-cell lymphoma, and it is currently in phase 1-2 trials for multiple liquid and solid malignancies (Chari et al., 2019). In addition to its antineoplastic properties, selinexor also has anti-inflammatory activity, and it has been shown to ameliorate LPS-induced lung injury in mice (Wu et al., 2018).

Herein we present an *in vitro* model to examine the effect of selinexor on neutrophil function. We demonstrate a significant inhibitory effect of selinexor on NETs formation, while other essential neutrophils processes are conserved. The results presented in this study may pave the way for the potential use of selinexor for the treatment of NETs-associated pathologies.

## Materials and methods

### Ethics statement

This study was conducted according to the principles expressed in the Declaration of Helsinki. All participating volunteers were informed and signed the consent form approved by the Institutional Review Board of the Tel Aviv Medical Center IRB 0502-19-TLV**.**

### Reagents

Selinexor (KPT330), was kindly provided by Karyopharm Therapeutics. Phosphate buffered saline (PBS) and Hanks’ balanced salt solution (HBSS) was obtained from Biological Industries. EDTA, bovine serum albumin (BSA), human albumin, phorbol 12-myristate 13-acetate (PMA) and Triton X-100 were all purchased from Sigma-Aldrich. Poly-L-lysine solution (0.01%) and buffered 4% formaldehyde solution were acquired from Merck.

### Isolation of neutrophils

Human peripheral blood samples (2–5 ml) in EDTA-coated vacutainer tubes (Greiner Bio-One) were obtained from healthy volunteers. Neutrophils were isolated using the EasySep Direct Human Neutrophil isolation kit (StemCell Technologies Inc.) by immunomagnetic negative selection according to the manufacturer’s instructions. The number of isolated neutrophils was quantified using Beckman coulter DxH800 hematology analyzer (Beckman Coulter Inc.) and the final concentration was adjusted to 10^7^/ml in RPMI without pH indicator.

### Neutrophil elastase enzymatic activity

10^5^ neutrophils were lysed in 0.2% Triton X-100 solution and incubated with chromogenic peptide elastase substrate at final concentration of 0.5 mM (stock of 20 mM in DMSO, Calbiochem) for 90 min at 37°C. Enzymatic activity was measured by an iMark Microplate Absorbance Reader (Bio-Rad) at 415 nm. A calibration curve was set up using purified NE between 5 and 100 µg (Athens Research and Technology). 10 µg of purified NE was used as positive control, and specific NE inhibitor IV at final concentration of 100 µM (stock of 20 mM in DMSO, Calbiochem) together with 10 µg of purified enzyme was used as negative control for each experiment.

### Myeloperoxidase enzymatic activity

10^5^ neutrophils were lysed in 0.2% Triton X-100 solution and incubated with of O-phenylenediamine at final concentration of 50 μg/ml (stock of 10 mg/ml in PBS, Sigma-Aldrich) and H_2_O_2_ at final concentration of 1 mM (Sigma-Aldrich) for 20 min at room temperature (RT). Enzymatic activity was measured by an iMark Microplate Absorbance Reader (Bio-Rad) at 450 nm. A calibration curve was set up by using purified MPO between 1 and 10 µg (Athens Research and Technology). 2 µg of purified MPO was used as positive control, and 4-aminobenzoic acid hydrazide (stock of 0.5 M in DMSO, Cayman Chemicals), a specific MPO inhibitor, at final concentration of 5 mM together with 2 µg of purified enzyme was used as negative control for each experiment.

### Oxidative burst assay by FACS

10^5^ neutrophils were added into FACS tubes in DHR-medium (HBSS with 0.1% human albumin and 1 mM EDTA). Catalase at the final concentration of 2000 U/ml (Sigma-Aldrich, 400 U/µl in DHR medium) was used to quench background signal and dihydrorhodamin 123 at final concentration of 25 µM (stock of 5 mM in DMSO, Invitrogen, DHR) were added and incubated for 10 min at 37°C. Then, neutrophils were activated with 1 µM PMA for 15 min at 37°C. Neutrophils without activation were used as negative control. Samples were immediately analyzed by FACSCanto II (Beckton Dickinson) and FlowJo V10 software.

### PAD4 inhibitory assay

The inhibitory effect of selinexor on PAD4 activity was examined using the PAD4 inhibitory screening assay kit (Cayman Chemicals) according to the manufacturer’s instructions. Human recombinant PAD4 enzyme provided in the assay kit was used together with specific PAD4 inhibitor Cl-amidine (final concentration of 100 µM) as positive control. Selinexor was used between 5 and 100 nM. All the experiments were performed in triplicate using the Synergy2 plate reader (BioTek) using a 360/40 nm fluorescent excitation filter and a 460/40 nm emission filter.

### Induction of NETs

2 × 10^5^ neutrophils were seeded on coverslips coated with poly-L-lysine and incubated with designated concentrations of selinexor (20 mM stock solution in DMSO, Karyopharm) for 2 h. Subsequently, cells were activated by the final concentration of 100 nM phorbol 12-myristate 13-acetate (PMA in DMSO, Sigma-Aldrich), 20 ng/ml transforming growth factor beta (TGF-β in 10 mM citric acid; Abcam), 20 ng/ml tumor necrosis factor-alpha (TNF-α in DDW; Peprotech), or 20 ng/ml interleukin 8 (IL-8 in DDW, Peprotech) for 3 h at 37°C and then fixed with 4% formaldehyde solution. RPMI complemented with DMSO used as control in case of 50 nM Selinexor. Additionally, 10^6^ neutrophils in 500 µl RPMI were placed in Eppendorf tubes and activated with 100 nM PMA for 3 h at 37°C. At the end of the incubation period, cells were collected by centrifugation at 3,000 rpm for 5 min and supernatant was collected for ELISA analysis.

### Immunofluorescent staining

Following activation, neutrophils were labeled with Sytox Green (Invitrogen) and Hoechst 33342 (Sigma-Aldrich) nuclear dyes, or using specific antibodies against NE (polyclonal rabbit anti-human NE, 1:1,000; Merck) and histone-3 (monoclonal mouse anti-human H3, 1:500; Abcam). Images were taken using an LSM700 Laser Scanning Confocal Fluorescence microscope (Zeiss). For each sample, three regions of interest containing 100–200 cells were evaluated and NETs formation was counted manually. Using Sytox Green (30 nM) and Hoechst 33342 (10 μg/ml) nuclear dyes, neutrophils not forming NETs were defined as those with compact DNA stained with both dyes. NETs-forming neutrophils were defined as those having diffuse DNA stained only with Sytox Green. Applying antibody staining NE and H3, neutrophils not forming NETs were defined as those exhibiting high intensity signal with NE (green) but low intensity signal with H3 (red). NETs-forming neutrophils were defined as those exhibiting high intensity NE (green) and H3 (red) signals, showing co-localization (yellow). The percentage of NETs was calculated as the ratio of NETs-forming neutrophils to total number of neutrophils (NETs-forming and non-forming neutrophils).

### NE-DNA complex ELISA

NETs formation was quantified by measuring the amount of NE-DNA complexes in the supernatant of activated neutrophils. ELISA for NE-DNA complexes was carried out as described previously with minor modifications (Kano et al., 2016). In brief, 96-well plates (Corning Incorporated) were first coated with the rabbit monoclonal anti-human NE antibody (1 μg/ml; Abcam) and incubated overnight at 4°C. Subsequently, 96-well plates were washed 3-times with PBS and incubated with blocking solution containing 1% bovine serum albumin in PBS for 90 min at RT. Next, supernatants of activated neutrophils (final dilution at 1:10) was added to 96-well plates and processed with a limited 15-min DNase digestion, in order to shorten chromatin threads for a maximum binding between NE-antibody and NE-DNA from NETs, before overnight incubation at 4°C. The following day, monoclonal mouse anti-human DNA antibody (Abcam) were applied to the wells for 90 min at RT, followed by Peroxidase AffiniPure Goat Anti-Mouse IgG (H + L) (Jackson ImmunoResearch Inc.) for 60 min at RT and then color development according to the manufacturer’s instructions. OD was measured for each well at a wavelength of 415 nm, using 490 nm as reference iMark Microplate Absorbance Reader (Bio-Rad).

### Statistical analysis

Shapiro-Wilk test was used for normality and no data was found to be normally distributed. Nonparametric Wilcoxon matched-pairs signed rank test or one-way ANOVA with Neuman-Keuls multiple comparison *post hoc* test were used to determine statistical differences using Graph Pad Prism 5 software. Statistical tests for comparison were one-tailed, and *p* < 0.05 was considered significant. Linear regression was used to calculate correlation data. Data are shown as box plots with the central line in the box representing the median, while the box indicating the full distribution of the data.

## Results

### Selinexor inhibits PMA-induced NETs formation *in vitro*

**FIGURE 1:**
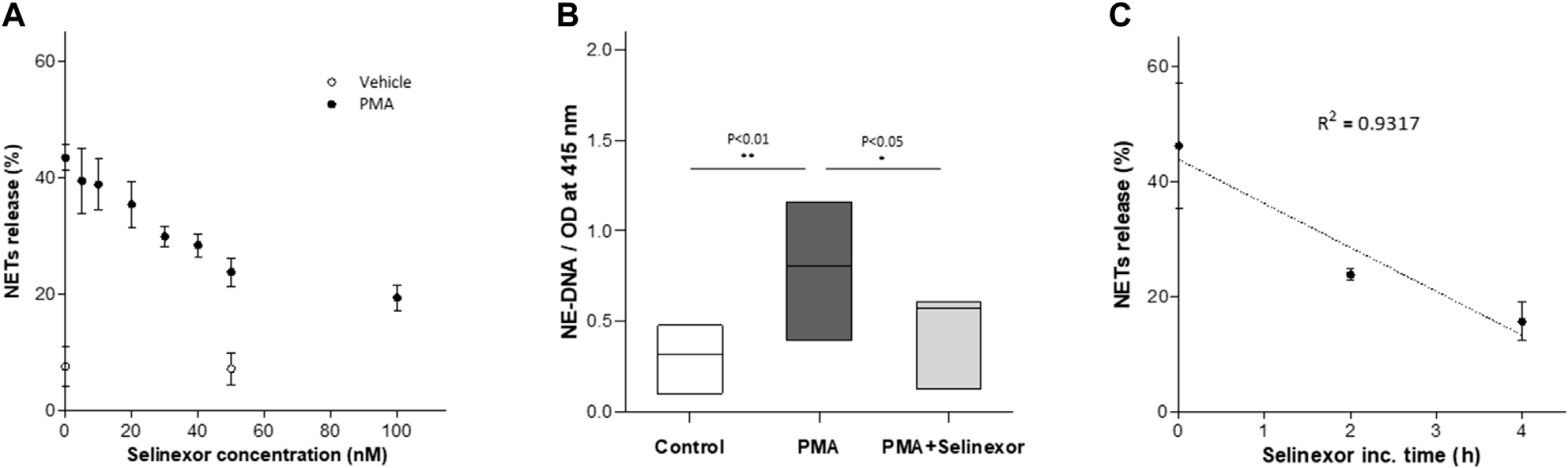
Selinexor inhibits NETs formation in a dose-dependent manner. Neutrophils were incubated with selinexor, at different concentrations, or a vehicle and then activated with PMA. NETs formation was assessed by confocal microscopy **(A)** Incubation with Selinexor at concentrations of 0–100 nM demonstrate that PMA-induced NETs formation 43.4% ± 2.2 (*N* = 8) was inhibited and plateaued at a concentration of 50 nM Selinexor (●, *N* = 8). Neutrophils incubated without PMA using 0 and 50 nM selinexor was used as control (○, *N* = 8). **(B)** NE-DNA complex ELISA assay demonstrating reduced NETs formation (*N* = 5, *p* < 0.01) following incubation with 50 nM Selinexor and PMA activation compared to PMA activation alone (OD 0.47 ± 0.09 vs. 0.83 ± 0.13 accordingly). Neutrophils incubated without PMA and selinexor were used as negative control and had a value of 0.30 ± 0.08. . **(C)** Positive correlation between selinexor incubation time and inhibition of NETs formation was observed (*R* ^2^ = 0.9317).

**FIGURE 2:**
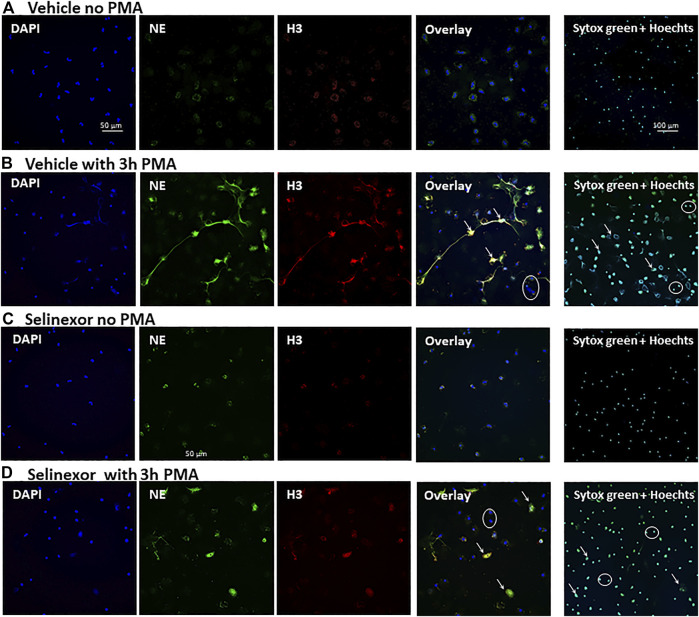
Inhibitory effect Selinexor on NETs formation, as seen in microscopy. Immunofluorescent (IF) staining demonstrating a significant inhibition of NETs formation following incubation of neutrophils with 50 nM Selinexor for 2 h vs. vehicle. Cells were activated with 100 nM PMA for 3 h and compared to non-activated cells. **(A)** Neutrophils were incubated with vehicle and were not activated. No NETs release was seen **(B)** Neutrophils were incubated with vehicle and activated with PMA. Significant NETs release was seen. **(C)** Neutrophils were incubated with Selinexor and were not activated. No NETs release was seen. **(D)** Neutrophils were incubated with Selinexor and activated with PMA. Minor NETs release was seen. Columns, from left to right: DAPI nuclear staining (blue), Neutrophil elastase (green), Histone 3 (red) and the Overlay of all three images. On the right columns Sytox green and Hoechst 33342 double staining. Representative NETs-forming neutrophils are indicated with white arrows, while neutrophils not forming NETs are signed with white circles.

Previous data describing the anti-inflammatory effects of selinexor suggests that this effect may be mediated by inhibition of neutrophil functions (Wu et al., 2018). We thus aimed to determine whether selinexor can inhibit NETs formation triggered by PMA, a potent inducer of NETs release (Tatsiy and McDonald, 2018a). Previous study showed that XPO1 function, inhibited by selinexor, is required for T cell development and function. Furthermore, selinexor concentration that allowed normal immune homeostasis was shown to be up to 100 nM (Tyler et al., 2017). Accordingly, neutrophils were incubated with selinexor at concentrations between 0 and 100 nM for 2 h prior to PMA activation. Our results demonstrated that selinexor inhibited NETs formation in a dose-dependent manner. The maximal inhibitory effect was achieved using 50 nM of selinexor, where NETs formation was 46.2% ± 4.0 and after selinexor treatment NETs reduced to 23.8 ± 2.4 exhibiting 49% inhibition (Figure 1A). Consequently, a concentration of 50 nM selinexor was used for all subsequent experiments. In addition to the gold-standard of fluorescent microscopy evaluation of NETs formation *via* co-localization of NE and H3 (Brinkmann et al., 2016) (Figure 2), we also applied a recently-developed method, NE-DNA complex ELISA (Kano et al., 2016), to quantify PMA-induced NETs formation in the presence or absence of selinexor. Incubation with 50 nM selinexor resulted in a 45% reduction of PMA-induced NETs release, as represented by OD 0.47 ± 0.09 compared to 0.83 ± 0.13 without selinexor, while negative control without PMA activation was 0.30 ± 0.08 (Figure 1B). Next, we sought to examine the effect of selinexor incubation time on NETs formation. The results demonstrate a mean inhibitory effect of 46.5% after 2 h and 68.4% following 4 h of selinexor treatment. A statistically significant positive correlation (*R* ^2^ = 0.9317) between selinexor incubation time and inhibition of NETs formation was observed (Figure 1C).

### NETs formation induced by several physiological neutrophil stimulators is inhibited by selinexor

**FIGURE 3:**
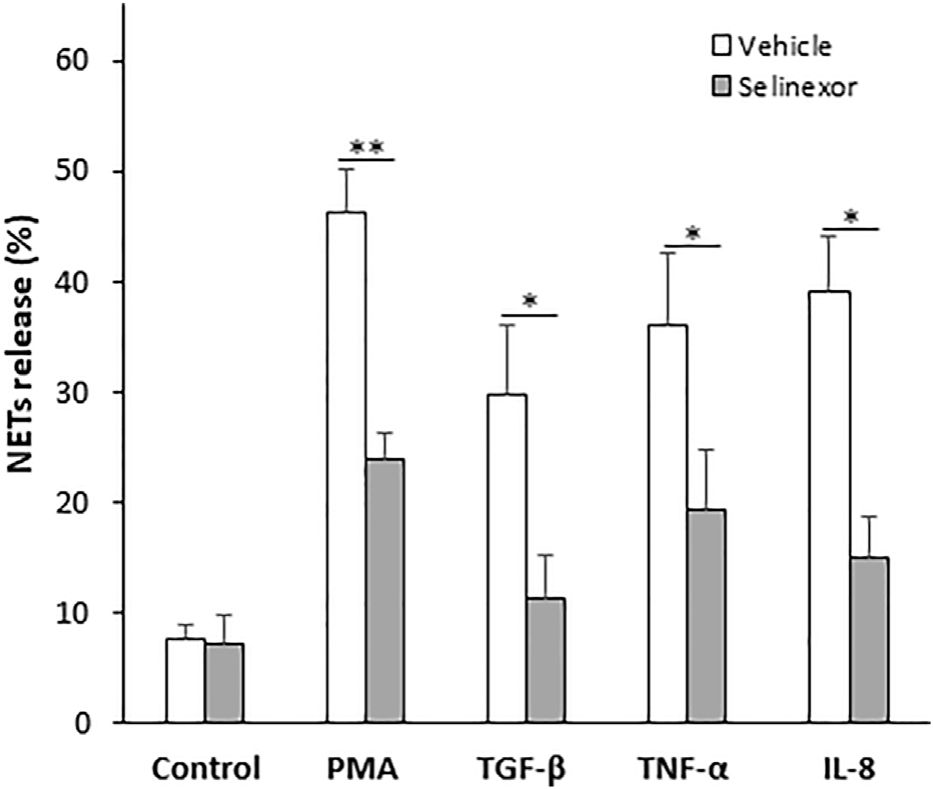
Selinexor inhibits NETs formation induced by various stimulators of neutrophils. Different activators, including 20 ng/ml TGF-β (*N* = 5), 20 ng/ml TNF-α (*N* = 5) and 20 ng/ml IL-8 (*N* = 5) induced NETs release compared to positive control using 100 nM PMA (*N* = 8). Neutrophils incubated without an inducer were used as negative control (*N* = 8). Incubation with Selinexor inhibited NETs release by the inducers in a different extent. In case of PMA the inhibitory effect was 48.5% and statistically significant (*p* < 0.01). In case of TGF-β, TNF-α and IL-8 selinexor’s inhibitory effect was 61.6%, 46.4%, and 61.3% respectively (*p* < 0.05).

NETs can form in the context of various pathological conditions, including infection, acute and chronic inflammation as well as neoplastic diseases. The cytokines that trigger NETs formation in these conditions vary (Kessenbrock et al., 2009; Megens et al., 2012; Lee et al., 2017; Mikacenic et al., 2018; Masucci et al., 2020; Middleton et al., 2020). Furthermore, the signal transduction that leads to NETs formation and the structure of the NETs themselves also can be different (Tatsiy and McDonald, 2018a). We thus sought to investigate whether selinexor inhibits NETs formation induced by several physiologically relevant neutrophil activators. We triggered NETs with inducers that are suggested to be involved in inflammatory and neoplastic conditions, such as IL-8 (Yang et al., 2020); TNF-α (Turner et al., 2014); and TGF-β (Fridlender et al., 2009). As a negative control, neutrophils were incubated without an inducer, while neutrophils incubated with PMA (100 nM) served as positive control. All the applied NETs-inducers triggered the release NETs, and selinexor inhibited NETs formation in all cases (Figure 3, Table 1). Maximal inhibitory effect was observed for NETs release induced by TGF-β, for which selinexor inhibited NETs release by 61.6% compared to controls (Table 1).

**TABLE 1:** NETs release induced by different inducers used in our study and the effect of Selinexor on NETs release.

NETs release %										
Samples	Control		PMA		TGF-β		TNF-α		IL-8	
** **	Vehicle	Selinexor	Vehicle	Selinexor	Vehicle	Selinexor	Vehicle	Selinexor	Vehicle	Selinexor
1	2.0	3.5	44.3	20.1	38.8	10.1	37.6	21.4	38.6	9.1
2	10.8	8.9	35.0	17.7	14.2	4.4	26.9	9.6	28.5	11.0
3	13.1	25.2	52.0	24.4	20.7	6.3	18.4	7.1	48.7	22.0
4	2.9	2.4	35.6	21.0	49.7	26.2	56.8	38.3	51.9	25.3
5	5.7	2.2	39.4	19.9	25.0	10.2	40.1	20.2	27.1	8.2
6	8.9	4.1	69.8	39.6	—	—	—	—	—	—
7	9.2	5.4	44.8	21.7	—	—	—	—	—	—
8	8.3	5.7	48.5	25.8	—	—	—	—	—	—
**Average of samples**	**7.5**	**7.4**	**45.8**	**23.5**	**29.7**	**11.4**	**36.0**	**19.3**	**39.0**	**15.1**
SE of samples	2.7	2.6	16.2	8.3	13.3	5.1	16.1	8.6	17.4	6.8
**Inhibition of Selinexor**		5.3		48.5		61.6		46.4		61.3

### Selinexor does not affect the main enzymatic processes that orchestrate NETs formation

**FIGURE 4:**
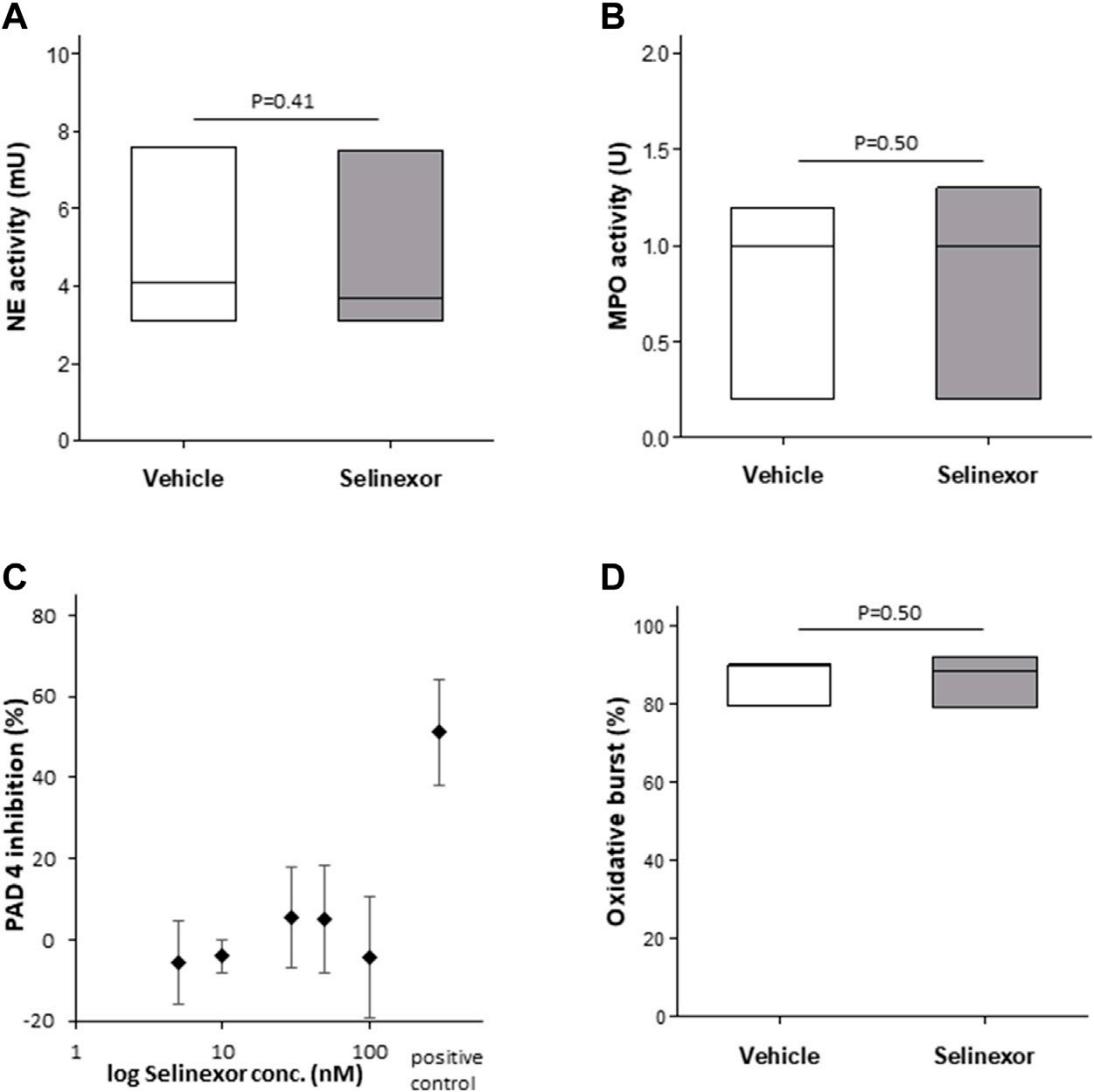
Selinexor does not affect the NETs formation machinery **(A)** Incubation of neutrophils with 50 nM Selinexor had no effect **(A)** on NE enzymatic activity (*N* = 3; *p* = 0.41); **(B)** MPO enzymatic activity (*N* = 3; *p* = 0.50) **(C)** PAD4 enzymatic activity (*N* = 3) and **(D)** oxidative burst (*N* = 3; *p* = 0.50).

We next aimed to investigate the mechanism by which Selinexor inhibits NETs formation. As the signal transduction that culminates in NETs formation has been partially unraveled (Metzler et al., 2014), we sought to investigate the effect of selinexor on enzymatic processes that are known to orchestrate NETs formation, including oxidative burst, NE (Baron et al., 2019), MPO and PAD4 enzymatic activities. For this purpose, we incubated neutrophils with 50 nM of selinexor or vehicle for 2 h prior to measurement of enzymatic activities. None of the enzymatic activities of NE, MPO and PAD4 were significantly influenced by 2 h-selinexor treatment (Figures 4A–C). In addition, we show no inhibitory effect of selinexor on PMA-induced oxidative burst (Figure 4D).

## Discussion

Chromatin externalization by neutrophils was described over 15 years ago in the context of host defense against infections. However, NETs formation has recently gained attention as a mechanism involved in a wide array of non-infections conditions. Given the deleterious effects of NETs in conditions such as autoimmunity, thrombus formations and cancer, the possibility that inhibition of NETs release might alter these pathologies is appealing. While some inhibitors that target enzymes participating in NETs formation have been described for pre-clinical use, none of these direct inhibitors have thus far been approved for use in humans. In a recent study, propofol used for sedation of mechanically ventilated adults was presented as a potential inhibitor of NETs formation (Meier et al., 2019). NE-specific inhibitor Sivelestat, an indirect NETs-inhibitor, is approved for clinical practice in Japan and South Korea (Aikawa and Kawasaki, 2014). In this brief research report, we reveal a drug that is approved for use in humans and can inhibit NETs release.

Selinexor, a specific inhibitor of XPO1 nuclear export protein is approved for cancer treatment. Animal experiments demonstrate that selinexor also has an anti-inflammatory effect that could be directed to rescue mice from LPS-induced lung injury (Wu et al., 2018). We speculated that some of the plethora of effects of selinexor could be attributed to inhibition on NETs formation, and this research was aimed to determine the effect of selinexor on this particular function of neutrophils. Using an *in vitro* model utilizing neutrophils from healthy donors, we assessed the effect of selinexor on NETs formation by fluorescent microscopy and NE-DNA complex ELISA, two of the common NETs assays. PMA-induced NETs formation was inhibited by selinexor in a dose-dependent manner, decreasing NETs release by over 50% with selinexor concentrations of 50 nM, which corresponds to the suggested clinical dosing (Tyler et al., 2017). We also expanded our investigation to physiological NETs-inducers: IL-8 a key mediator associated with inflammation (Yang et al., 2020); TNF-α is a prominent inflammatory cytokines involved in various auto-inflammatory conditions (Turner et al., 2014); and TGF-β activates neutrophils in cancer (Fridlender et al., 2009). These physiologic activators induced NETs to different extents, and in all the conditions selinexor inhibited NETs release at least by 50%, with the highest effect seen in the TGF-β stimulation group. Inducers of NETs release pose their effect *via* intra-cellular pathways that culminate in NE release from neutrophil granules (Metzler et al., 2014) and PAD4 citrullination of histones (Lewis et al., 2015). Interestingly, our experiments show that Selinexor does not affect the basic components of the NETs-forming machinery, which is in line with the fact Selinexor treatment is not associated with increased rate of infections in MM patients treated with Selinexor (Abid et al., 2021). These results may have several explanations. The drug might exert its action either on kinases (i.e., TAK1, p38 MAPK, MEK) controlling the early events of NETs formation process or on the later phases of NETs release following the action of the investigated enzymes (Lapponi et al., 2013; Tatsiy and McDonald, 2018b; Morales-Primo et al., 2022). Alternatively, selinexor might interfere with localizing of the enzymes to the designated cellular compartment (nucleus or cytoplasm) in the correct timing for NETs to be released. Further study will examine the effect of selinexor on the intracellular spatial localization of NET machinery and evaluate its effect on relevant pathways.

The *in vitro* inhibitory effect of selinexor on NETs formation is repetitive and significant. However, the effect of selinexor on intra- and extra-vascular NETs is not defined *in vivo*. Hence, further *in vivo* study is needed to investigate the potential of selinexor for inhibition of NETs formation and its clinical significance in reversal of NETs-associated pathologies.

In conclusion, here we show that an oral drug that is approved for use in humans can significantly inhibit NETs release *in vitro*. These findings might pave the way for the potential use of selinexor in disease in which NETs formation plays a role in disease pathogenesis, in an overall effort to reduce disease burden.

## Data Availability

The original contributions presented in the study are included in the article/Supplementary Materials, further inquiries can be directed to the corresponding author.
