# Supplementary material for: Selinexor, a selective inhibitor of nuclear export, inhibits human neutrophil extracellular trap formation in vitro
Source: Front Pharmacol. 2022 Nov 24;13:1030991. doi: 10.3389/fphar.2022.1030991 (PMC9730241; doi:10.3389/fphar.2022.1030991)
Supplement: Supplementary file 1 [file Table1.docx]

| **NETs release %** | | | | | | | | | | |
| --- | --- | --- | --- | --- | --- | --- | --- | --- | --- | --- |
| **Samples** | **Control** | | **PMA** | | **TGF-β** | | **TNF-α** | | **IL-8** | |
|  | Vehicle | Selinexor | Vehicle | Selinexor | Vehicle | Selinexor | Vehicle | Selinexor | Vehicle | Selinexor |
| 1 | 2.0 | 3.5 | 44.3 | 20.1 | 38.8 | 10.1 | 37.6 | 21.4 | 38.6 | 9.1 |
| 2 | 10.8 | 8.9 | 35.0 | 17.7 | 14.2 | 4.4 | 26.9 | 9.6 | 28.5 | 11.0 |
| 3 | 13.1 | 25.2 | 52.0 | 24.4 | 20.7 | 6.3 | 18.4 | 7.1 | 48.7 | 22.0 |
| 4 | 2.9 | 2.4 | 35.6 | 21.0 | 49.7 | 26.2 | 56.8 | 38.3 | 51.9 | 25.3 |
| 5 | 5.7 | 2.2 | 39.4 | 19.9 | 25.0 | 10.2 | 40.1 | 20.2 | 27.1 | 8.2 |
| 6 | 8.9 | 4.1 | 69.8 | 39.6 |  |  |  |  |  |  |
| 7 | 9.2 | 5.4 | 44.8 | 21.7 |  |  |  |  |  |  |
| 8 | 8.3 | 5.7 | 48.5 | 25.8 |  |  |  |  |  |  |
| **Average of samples** | **7.5** | **7.4** | **45.8** | **23.5** | **29.7** | **11.4** | **36.0** | **19.3** | **39.0** | **15.1** |
| SE of samples | 2.7 | 2.6 | 16.2 | 8.3 | 13.3 | 5.1 | 16.1 | 8.6 | 17.4 | 6.8 |
| **Inhibition of Selinexor** |  | 5.3 |  | 48.5 |  | 61.6 |  | 46.4 |  | 61.3 |

**Supplementary Table 1. NETs release induced by different inducers used in our study and the effect of Selinexor on NETs release**
